# Influence of polymorphisms of the beta-2 adrenergic receptor on the presence of exercise-induced bronchospasm in adolescents✰

**DOI:** 10.1016/j.rppede.2015.06.021

**Published:** 2016

**Authors:** Cássio Leandro Mühe Consentino, Lupe Furtado-Alle, Larissa Rosa da Silva, Wendell Arthur Lopes, Luciane Viater Tureck, Gerusa Einsfeld Milano, Leilane Lazarotto, Cláudia Regina Cavaglieri, Neiva Leite

**Affiliations:** a Universidade Federal do Paraná (UFPR), Curitiba, PR, Brazil; b Universidade Estadual de Maringá (UEM), Ivaiporã, PR, Brazil; c Universidade Estadual de Campinas (Unicamp), Campinas, SP, Brazil

**Keywords:** Adolescent, Exercise-induced bronchospasm, ADRB2 gene

## Abstract

**Objective:**

To determine the influence of polymorphisms of the beta-2 adrenergic receptor (ADRB2) in triggering exercise-induced bronchospasm (EIB) in adolescents.

**Methods:**

The subjects were divided into two groups: present EIB (EIB+) (n=45) and absent EIB (EIB−) (n=115). The bronchial provocation test with exercise was performed with a protocol that consisted of walking/running for at least eight minutes at high intensity, i.e., >85% of maximum heart rate, considering EIB+ as a 10% decrease in forced expiratory volume in one second (FEV_1_). The genotyping of the *ADRB2* gene was performed by the Taqman method, using the Step One Plus system. Independent *t*-test, Mann–Whitney and Chi-square tests, as well as Spearman's correlation coefficient were used for the statistical analysis.

**Results:**

Age, body weight, height, FEV_1_, FVC and FEV_1_/FVC ratio were lower in the EIB+ group when compared to EIB− (*p*<0.05). There were no significant differences in the proportion of the allele at position 27 and *Arg16Gly* and *Gln27Glu* genotypes between the EIB+ and EIB− groups (*p*=0.26; *p*=0.97 and *p*=0.43, respectively). However, there was a trend toward statistical significance regarding the greater proportion of the Gly16 allele for the EIB+ when compared to the EIB− group (*p*=0.08).

**Conclusions:**

The presence of polymorphisms associated with the Glu27 allele and *Arg16Gly* and *Gln27Glu* genotypes had no influence on EIB. However, the statistical trend toward greater frequency of the *Gly16* allele in individuals with EIB+ can be considered evidence of the influence of polymorphisms of the *ADBR2* gene on EIB in adolescents.

## Introduction

Exercise-induced bronchospasm (EIB) is defined as temporary narrowing of the airways that occurs after strenuous exercise in up to 90% of asthmatic individuals^1^ and in almost 20% of individuals with no history of respiratory disease.^2^ The presence of excess weight can contribute to increased severity of EIB in asthmatics.^3^ The excessive accumulation of fatty tissue in the central region can change the pulmonary mechanics and airway inflammatory response, leading to increased contractility and responsiveness of bronchial smooth muscle^4^ and thus limit the practice of physical exercises^5^ as therapy for asthma^6^ and obesity.^7^

Some genetic alterations, such as polymorphisms of the beta adrenergic receptor 2 (*ADRB2*), have been associated with the presence of asthma^8^ and obesity.^9^ The *ADRB2* gene is located on chromosome 5q31 and can be found in several regions of the body, including smooth muscle.^10^ *ADRB2* act through mediation by adrenaline and noradrenaline action and promote smooth muscle relaxation, even in the pulmonary region,^11^ as well as playing an important role in bronchodilation during exercise in healthy individuals.^12^ The *Arg16Gly* and *Gln27Glu* polymorphisms of the *ADRB2* gene have been associated with asthma symptoms,^7^ including reduction in the pulmonary function and in the bronchodilation response to medication, as they have negative influence on the bronchodilator effect,^13^ a therapeutic resource that is part of pre-exercise EIB prevention.^14^

Recently, our research group found a higher presence of *Arg16Gly* polymorphism in children and adolescents with asthma when compared with controls. Additionally, there was a trend for a higher frequency of polymorphism *Gly16* in asthmatics with excess weight.^15^ However, the influence of the polymorphism presence on the *ADRB2* receptor in the presence of EIB in children and adolescents has not been investigated. Our hypothesis is that the higher frequency of polymorphisms in *ADRB2* receptor could be related to higher presence of EIB in this population. Therefore, the aim of this study was to determine the influence of polymorphisms in the *ADRB2* gene on the triggering of EIB in adolescents.

## Method

This was a cross-sectional study of 160 adolescents of both genders, of Caucasian ethnicity, aged between 9 and 17 years, selected for convenience and from public schools in the city of Curitiba, state of Parana, Brazil. The sample was divided in two groups, with EIB (EIB+) (n=45) and without EIB (EIB−) (n=115). The presence of EIB was verified when there was a decrease ≥10% in FEV_1_ in relation to the baseline value at the bronchial provocation test through exercise. All participants and parents/tutors signed the free and informed consent form, according to the research project approved by the Ethics Committee on Human Research of Hospital de Clinicas of Universidade Federal do Parana (protocol n. 2460.067/2011-03). Sample size calculation was performed with a 95% confidence level and the formula described by Santos.^16^ The size of the calculated sample was of 246 students. However, the number of participants comprised 160 adolescents, 65% of the expected sample, due to the complexity of the tests and the need for blood collection.

Body weight (kg) was measured on a digital scale (Toledo^®^) with 0.1kg resolution, and height (cm) in a stadiometer (Sanny^®^) with a resolution of 0.1cm. The body mass index (BMI) was calculated using the formula: BMI (kg/m^2^)=body mass (kg)/height^2^ (m). This variable was converted to BMI *z*-score, using the WHO Anthroplus software v.1.0.4 developed by the World Health Organization (WHO), classified according to the cutoff points proposed by the WHO in 2006.^17^

Waist circumference (WC) was measured in centimeters (cm) using an inextensible anthropometric tape (Cardiomed^®^), measured at midpoint between the last rib and the iliac crest, with the individual in the standing position, relaxed abdomen and arms positioned along the body. The classification used the values proposed by Fernández et al. in 2004.^18^

The diagnosis of asthma, according to the III Brazilian Consensus on Asthma Management (SBPT, 2002)^19^ was performed using medical assessment and the International Study of Asthma and Allergies in Childhood (ISAAC) questionnaire and the disease was confirmed through the answer to question number 6.

Pulmonary function was assessed by spirometry in the pre-exercise and post-exercise (5, 10 and 15min). They were made three maneuvers with the evaluated in a sitting position and nose clip. The curves were selected that showed the highest values for the variables of forced expiratory volume in one second (FEV_1_) and forced vital capacity (FVC) in liters (L). The predicted values and the forced expiratory coefficient (FEV_1_/FVC) were shown by the spirometer software (One brand Flow^®^), using as reference values those proposed by Knudson et al.^20^

The bronchial provocation test with exercise was carried out in a controlled environment (temperature between 20 and 25°C and relative humidity of 50%), in the afternoon, on a treadmill (Master Super ATL-Inbramed) with a protocol that consisted in walking/running for eight minutes at an intensity >85% of maximum heart rate (HR_max_), according to the American Thoracic Society guidelines.^14^ HR_max_ was calculated using the formula proposed by Tanaka et al.^21^ and monitored during the test using a heart rate meter (Polar^®^). The assessed subjects were instructed not to drink caffeine-based drinks two hours before the evaluation, discontinue use of short and long-action bronchodilators 48h and 5 days before the evaluations, respectively.

The exercise was not performed when there were reports of asthma crisis or viral infection of the airways in the 4 weeks before the test. The magnitude of the decrease was calculated from the maximum decrease in FEV_1_ (MDVEF_1_) through the equation: MDFEV_1_=[(pre-exercise FEV_1_−lowest post-exercise FEV_1_)/pre-exercise FEV_1_]×100.^14^ DNA was extracted from blood samples and genotyping polymorphisms *Arg16Gly* and *Gln27Glu* of *ADRB2* gene by performed through the Taqman method, using a TaqMan SNP genotyping assay kit of Applied Biosystems and Eppendorf realplex v.1.5 software, with the Step One Plus equipment. Next, a scatter plot (XY) was made for the FAM-VIC separation and subsequent genotyping of each adolescent for each polymorphism. The individuals homozygous for the amino acid arginine at codon 16 (*ArgArg*) and glutamine at codon 27 (*GlnGln*) were classified as normal, whereas individuals homozygous at position 16 for the amino acid glycine (*Gly*) and at position 27, for glutamic acid (*GluGlu*) were classified as carriers, as well as the heterozygous individuals.

Statistical analysis was performed using SPSS software, version 19. Normality was verified using the Kolmogorov–Smirnov test, Student's *t* test was applied in parametric variables to compare the groups, whereas the Mann–Whitney *U* test was applied on non-parametric ones. The chi-square test was used to analyze the proportions between the groups. The correlation between variables was assessed using Spearman's correlation coefficient and classified according to Dancey and Reidy.^22^ The significance level was set at *p*<0.05.

## Results

The baseline characteristics of the groups are shown in Table 1. Differences were observed for the variables age, body weight, height, FEV_1_ (both in liters and percentage of predicted value) and FVC in liters and in the FEV_1_/FVC ratio, being lower in the EIB+ group.

**Table 1:** Anthropometric and baseline spirometric characteristics of the groups present (+) and absent (−) exercise-induced bronchospasm.

Variables	EIB+ (n=45)	EIB− (n=115)	*t* or *U*	*p* -value
Age (years) a	13.6±1.6	14.5±1.5 **	4.05	0.00
Body mass (kg)	65.0±15.2	74.5±18.3 **	−3.09	0.00
Height (cm)	160.4±9.4	165.5±8.9 **	−3.18	0.00
BMI (kg/m^2^)	25.3±5.5	27.0±5.3	−1.88	0.06
BMI *z* -score	1.6±1.3	1.9±1.2	−1.30	0.19
WC (cm)	84.6±13.6	84.9±13.9	1.57	0.12
VEF_1_ (L)	2.9±0.5	3.4±0.6 **	−4.28	0.00
FEV_1_ (% predicted)	95.4±10.8	102.0±18.8 *	−2.21	0.02
FVC (L)	3.4±0.7	3.9±0.8 **	−3.57	0.00
FVC (% predicted)	102.3±12.2	106.0±13.4	−1.61	0.10
FEV_1_/FVC (%) a	86.7±10.3	88.5±7.0 *	2.01	0.04

* *p* <0.05.

** *p* <0.01.

a Variables without normal distribution.

BMI, body mass index; WC, waist circumference; FEV_1_, forced expiratory volume in one second; FVC, forced vital capacity; FEV_1_/FVC, FEV_1_/FVC ratio.Values expressed in mean±standard deviation.

Regarding the polymorphisms of the *ADRB2* gene, no significant differences were found for allele 27 and for genotypes of *Arg16Gly* and *Gln27Glu* polymorphism between the EIB+ and EIB− groups (*p*=0.26; *p*=0.97 and *p*=0.43, respectively). However, there was a trend toward statistical significance for a greater proportion of polymorphisms at allele 16 in the EIB+ group when compared to the EIB− (*p*=0.08) (Table 2).

**Table 2:** Frequency of alleles and genotypes of *ADRB2* gene between the groups present (+) and absent (−) exercise-induced bronchospasm.

		EIB+	EIB−	*X* ^2^	*p* -value
*Alleles*					
	Arg	30 (37.5%)	103 (48.6%)	2.87	0.08
	Gly	50 (62.5%)	109 (51.4%)		
	Total	80 (100%)	212 (100%)		
	Gln	47 (69.1%)	140 (69.3%)	0.001	0.97
	Glu	21 (30.9%)	62 (30.7%)		
	Total	68 (100%)	202 (100%)		

*Genotypes*					
	ArgArg	7 (17.5%)	28 (26.4%)	2.64	0.26
	ArgGly	16 (40.0%)	47 (44.4%)		
	GlyGly	17 (42.5%)	31 (29.2%)		
	Total	40 (100%)	106 (100%)		
	GlnGln	19 (55.9%)	51 (50.5%)	1.68	0.43
	GlnGlu	9 (26.5%)	38 (37.6%)		
	GluGlu	6 (17.6%)	12 (11.9%)		
	Total	34 (100%)	101 (100%)		

*Arg* , arginine; *Gly* , glycine; *Gln* , glutamine; *Glu* , glutamic acid; values expressed in absolute and relative frequencies.

The percentage of maximum FEV_1_ decrease showed a moderate correlation with the presence of asthma (rho=0.47; *p*<0.01). However, for the variables BMI *z*-score (rho=0.01), WC (rho=0.20), polymorphisms *Arg16Gly* (−0.01) and *Gln27Glu* (rho=−0.07) showed no significant correlations.

## Discussion

The aim of this study was to determine the influence of polymorphisms in the *ADRB2* gene on the triggering of EIB in adolescents. There were no significant differences in frequencies for allele 27 and for the genotypes of allele *Arg16Gly* and *Gln27Glu* in the EIB+ group compared to the EIB−, which partly refutes our initial hypothesis. However, there was a trend toward statistical significance for a higher frequency of allele 16 in the EIB+ group when compared to the EIB−. This finding may be regarded as evidence of the association between polymorphisms in the *ADRB2* gene and the presence of EIB.

Individuals diagnosed with EIB+ have reduced pulmonary function compared to EIB− individuals, except for FVC (% of predicted), which did not differ between the two groups.^23^

On the other hand, previous studies did not identify such differences when assessing obese adolescents,^3^ and obese asthmatics^24^ and obese individuals with rhinitis.^25^ These divergences may be explained by differences in age and initial height of the groups in the present study.

The percentage of maximum FEV_1_ decrease showed moderate correlation with a history of asthma, which differs from the results found by Cichalewski et al.^26^ The methodological differences in the diagnosis of EIB can explain this discrepancy, as the present study used a bronchial provocation test on treadmill and in the study of Cichalewski et al.,^26^ it was performed 45min after a physical education class. On the other hand, neither study identified an association between EIB and BMI.

No previous studies were found to assess the frequency of *ADRB2* polymorphisms in individuals with and without EIB. In this study, the frequency of allele *Gly16* was 62.5% and 30.7% for allele Glu27. The proportion of the first was higher than the one found in a study in asthmatics, which was 46.6%.^8^ However, it is similar to the findings for the general population (61%).^27^ The frequency of allele *Glu27* was similar between the studies.^8^^,^ 27

A study carried out by Snyder et al.^28^ observed in healthy adults that individuals with the *Arg16Arg* and *Gly16Gly* genotypes had similar responses to bronchodilation during exercise. However, after the end of the test, individuals that were homozygous for allele *Arg16* returned more quickly to baseline when compared to *Gly16* homozygotes. The authors explain this finding by a possible desensitization of the *ADRB2* gene in this population. These results differ from those found in this study, which showed no differences in frequency between the genotypes and can actually confirm that there is no influence of this gene in individuals with EIB.

The polymorphisms of *ADRB2* gene might be related to asthma, mainly due to being associated with increased airway sensitivity.^8^ Bronchial hyper-responsiveness is one of the main characteristics of asthma and may be triggered by several factors, including exercise.^29^ The study carried out by Fukui et al.^30^ showed that individuals with increased responsiveness to methacholine challenge test had a polymorphism at codon 16. EIB can be considered an exaggerated airway response.^1^ Thus, it was expected that polymorphisms had an effect on the bronchoconstrictor response to exercise, which ultimately did not occur.

The cross-sectional study design limits the causal associations between the variables. Another issue to be emphasized as a limiting factor is the low number of participants for the analysis of genetic polymorphisms, leading to a cautious interpretation of the study findings. We suggest studies with larger sample sizes to confirm the association between the presence of the *Gly*16 allele and the manifestation of EIB. Further studies are required with the control of the aforementioned limitations and the use of spirometry at times 3; 5; 10; 15 and 30min after bronchial provocation challenge tests to prevent possible EIB misdiagnosis.

We conclude that the presence of polymorphisms associated with *Glu27* allele and *Arg16Gly* and *Gln27Glu* genotypes did not influence EIB expression. However, the statistical trend for increased frequency of allele *Gly16* in individuals with EIB can be considered an indication of the influence of polymorphisms on the gene *ADBR2* in EIB in adolescents.
