# Supplementary material for: Influence of polymorphisms of the beta-2 adrenergic receptor on the presence of exercise-induced bronchospasm in adolescents
Source: Rev Paul Pediatr. 2016 Jan-Mar;34(1):24–9. doi: 10.1016/j.rppede.2015.06.021 (PMC4795718; doi:10.1016/j.rppede.2015.06.021)
Supplement: Translation

# Translation: Influência dos polimorfismos no receptor beta 2 adrenérgico na presença de broncoespasmo induzido pelo exercício em adolescentes✰

**Authors:** Cássio Leandro Mühe Consentino, Lupe Furtado-Alle, Larissa Rosa da Silva, Wendell Arthur Lopes, Luciane Viater Tureck, Gerusa Einsfeld Milano, Leilane Lazarotto, Cláudia Regina Cavaglieri, Neiva Leite

**Affiliations:** a Universidade Federal do Paraná (UFPR), Curitiba, PR, Brasil; b Universidade Estadual de Maringá (UEM), Ivaiporã, PR, Brasil; c Universidade Estadual de Campinas (Unicamp), Campinas, SP, Brasil

**Keywords:** Adolescente, Broncoespasmo induzido pelo exercício, Gene *ADRB2*

## Abstract

**Objetivo:**

Determinar a influência dos polimorfismos dos receptores adrenérgicos beta 2 (*ADRB2*) no desencadeamento de broncoespasmo induzido pelo exercício (BIE) em adolescentes.

**Métodos:**

Os sujeitos foram divididos em dois grupos: BIE presente (BIE+) (n=45) e BIE ausente (BIE−) (n=115). O teste de broncoprovocação com exercício foi feito com protocolo que consistiu em caminhar/correr durante no mínimo oito minutos em intensidade superior a 85% da frequência cardíaca máxima, considerando como BIE presente uma queda de 10% do volume expiratório forçado no primeiro segundo (VEF_1_). A genotipagem do gene *ADRB2* foi feita pelo método Taqman por meio do aparelho Step One Plus. Para análise estatística usaram-se os testes *t* independente, U de Mann-Whitney, qui-quadrado e coeficiente de correlação de Spearman.

**Resultados:**

Idade, massa corporal, estatura, VEF_1_, CVF e relação VEF_1_/CVF foram menores no grupo BIE+ em comparação com o BIE− (*p*<0,05). Não houve diferenças significativas na proporção do alelo na posição 27 e dos genótipos *Arg16Gly* e *Gln27Glu* entre os grupos BIE+ e BIE− (*p*=0,26; *p*=0,97 e *p*=0,43, respectivamente). Entretanto, verificou-se uma tendência à significância estatística na maior proporção do alelo *Gly16* para o grupo BIE+ comparado com o BIE− (*p*=0,08).

**Conclusões:**

A presença de polimorfismos associados ao alelo *Glu27* e os genótipos *Arg16Gly* e *Gln27Glu* não influenciam no BIE. Porém, a tendência estatística observada para uma maior frequência do alelo *Gly16* nos indivíduos com a presença de BIE pode ser considerado indício da influência de polimorfismos no gene *ADBR2* no BIE em adolescentes.

## Introdução

O broncoespasmo induzido pelo exercício (BIE) é definido como estreitamento transitório das vias aéreas que acontece logo após exercícios intensos em até 90% dos indivíduos asmáticos^1^ e em quase 20% das pessoas sem histórico de doenças respiratórias.^2^ A presença de excesso de peso pode contribuir para aumento da gravidade do BIE em asmático.^3^ O acúmulo excessivo de tecido adiposo na região central pode alterar a mecânica pulmonar e a resposta inflamatória das vias aéreas, levar ao aumento da contratilidade e da responsividade da musculatura lisa dos brônquios^4^ e limitar assim a prática de exercícios físicos^5^ como terapêutica na asma^6^ e na obesidade.^7^

Algumas alterações genéticas, como os polimorfismos nos receptores adrenérgicos beta 2 (*ADRB2*), têm sido associadas à presença de asma^8^ e de obesidade.^9^ O gene do *ADRB2* está localizado no cromossomo 5q31 e pode ser encontrado em diversas regiões do corpo, inclusive na musculatura lisa.^10^ Os *ADRB2* atuam mediados pela ação da adrenalina e da noradrenalina e promovem o relaxamento do músculo liso, até na região pulmonar,^11^ além de desempenhar papel importante na broncodilatação durante o exercício em indivíduos saudáveis.^12^ Os polimorfismos *Arg16Gly* e *Gln27Glu* do gene do *ADRB2* vêm sendo relacionados a sintomas da asma,^7^ incluindo a redução da função pulmonar e da resposta broncodilatadora à medicação, pois têm influência negativa no efeito da medicação broncodilatadora,^13^ recurso terapêutico que faz parte da prevenção do BIE pré-exercício.^14^

Recentemente, nosso grupo de pesquisas verificou maior presença de polimorfismo *Arg16Gly* em crianças e adolescentes asmáticos em comparação com os controles. Além disso, verificou-se tendência a maior frequência do polimorfismo *Gly16* em asmáticos com excesso de peso.^15^ Contudo, a influência da presença de polimorfismo no receptor *ADRB2* na presença de BIE em crianças e adolescentes ainda não foi investigada. Nossa hipótese é que a maior frequência de polimorfismo no receptor *ADRB2* poderia estar relacionada à maior presença de BIE nessa população. Portanto, o propósito do presente estudo foi determinar a influência dos polimorfismos no gene *ADRB2* no desencadeamento de BIE em adolescentes.

## Método

Estudo transversal com 160 adolescentes de ambos os sexos, de etnia branca, entre 9-17 anos, selecionados por conveniência e provenientes de escolas públicas de Curitiba (PR). A amostra foi dividida em dois grupos, BIE presente (BIE+) (n=45) e BIE ausente (BIE−) (n=115). Constatou-se presença de BIE quando houve queda maior ou igual a 10% do VEF_1_ em relação ao valor basal no teste de broncoprovocação por exercício. Todos os participantes e responsáveis assinaram o termo de consentimento livre e esclarecido, conforme projeto de pesquisa aprovado pelo Comitê de Ética em Pesquisa em Seres Humanos do Hospital de Clínicas da Universidade Federal do Paraná (protocolo n° 2460.067/2011-03). O cálculo amostral foi feito com nível de confiança de 95% e a fórmula descrita por Santos.^16^ O tamanho da amostra calculado foi de 246 escolares. Entretanto, o número de participantes foi de 160 adolescentes, 65% da amostra prevista, em função da complexidade dos exames e da necessidade de coleta sanguínea.

Mensurou-se a massa corporal (kg) em balança digital (Toledo^®^), com resolução de 0,1kg, e a estatura (cm) em estadiômetro (Sanny^®^) com resolução de 0,1cm. O Índice de Massa Corporal (IMC) foi calculado pela fórmula IMC (kg/m^2^)=Massa corporal (kg)/Estatura^2^ (m). Essa variável foi convertida para IMC-escore Z, por meio do *software* WHO Anthroplus v.1.0.4 desenvolvido pela Organização Mundial da Saúde (OMS), classificado de acordo com os pontos de corte propostos pela OMS em 2006.^17^

A circunferência abdominal (CA) foi mensurada em centímetros (cm) com fita antropométrica inextensível (Cardiomed^®^), medida no ponto médio entre o último arco costal e a crista ilíaca, com o indivíduo de pé, abdome relaxado e braços ao longo do corpo. Para classificação, usaram-se os valores propostos por Fernandéz *et al*., em 2004.^18^

Para o diagnóstico da asma, conforme o III Consenso Brasileiro do Manejo da Asma (SBPT, 2002),^19^ usaram-se a avaliação médica e o questionário do International Study of Asthma and Allergies in Childhood (Isaac) e confirmou-se a doença por meio da questão de número 6.

A função pulmonar foi avaliada por espirometria no pré-exercício e pós-exercício (5, 10 e 15 minutos). Fizeram-se três manobras, com o avaliado em posição sentada e com clipe nasal. Foram selecionadas as curvas que apresentaram os maiores valores para as variáveis de volume expiratório forçado no primeiro segundo (VEF_1_) e capacidade vital forçada (CVF) em litros (L). Os valores preditos e o coeficiente expiratório forçado (VEF_1_/CVF) foram mostrados pelo *software* do espirômetro (marca One Flow^®^), que usa como referência os valores propostos por Knudson *et al*.^20^

O teste de broncoprovocação com exercício foi feito em ambiente controlado (temperatura entre 20-25°C e umidade relativa de 50%), no período da tarde, em esteira ergométrica (modelo Master Super ATL-Inbramed), com um protocolo que consistiu em caminhar/correr durante oito minutos, em intensidade superior a 85% da frequência cardíaca máxima (FC_máx_), de acordo com o guia da American Thoracic Society.^14^ A FC_máx_ foi calculada por meio da fórmula proposta por Tanaka *et al*.^21^ e monitorada, durante o teste, por frequencímetro (Polar^®^). Os avaliados foram orientados a não ingerir bebidas à base de cafeína, duas horas antes da avaliação, suspender o uso de broncodilatadores de ação curta e longa, 48 horas e cinco dias antes das avaliações, respectivamente. O exercício não foi feito quando houve relato de crise de asma ou infecção viral nas vias aéreas nas últimas quatro semanas antes do teste. A intensidade da queda foi calculada pela queda máxima do VEF_1_ (QMVEF_1_) por meio da equação: QMVEF_1_=[(VEF_1_ pré-exercício-VEF_1_ pós-exercício mais baixo)/VEF_1_ pré-exercício]×100.^14^

A extração do DNA ocorreu por meio de amostra sanguínea, com a genotipagem dos polimorfismos *Arg16Gly* e *Gln27Glu* do gene *ADRB2* pelo método *Taqman*, por meio do kit *TaqMan SNP genotyping assays* da Applied Biosystems e do *software Eppendorf realplex v.1.5*, com o uso do aparelho de Step One Plus. A seguir, foi feito um gráfico de dispersão (XY) para a separação FAM-VIC e consequente determinação do genótipo de cada adolescente para cada polimorfismo. Classificaram-se como usuais os indivíduos homozigóticos para o aminoácido arginina no códon 16 (*ArgArg*) e glutamina no códon 27 (*GlnGln*) e como portadores os indivíduos homozigóticos, na posição 16, para o aminoácido glicina (*GlyGly*) e, na posição 27, para ácido glutâmico (*GluGlu*), bem como os heterozigóticos.

A análise estatística foi feita no software SPSS versão 19. A normalidade foi verificada pelo teste de Kolmogorov-Smirnov. Para comparação dos grupos, aplicou-se o teste *t* de Student nas variáveis paramétricas e o teste U de Mann-Whitney nas não paramétricas. O teste qui-quadrado foi usado para analisar as proporções entre os grupos. A correlação entre as variáveis foi avaliada pelo coeficiente de correlação de Spearman e classificadas de acordo com Dancey e Reidy.^22^ O nível de significância adotado foi de *p*<0,050.

## Resultados

As características basais dos grupos são mostradas na tabela 1. Foram encontradas diferenças nas variáveis idade, massa corporal, estatura, VEF_1_ (tanto em litros quanto no percentual do predito) e CVF em litros e na relação VEF_1_/CVF, menores no grupo BIE+.

**Tabela 1:** Características antropométricas e espirométricas basais dos grupos broncoespasmo induzido pelo exercício (BIE) presente (+) e ausente (−)

Variáveis	BIE+ (n=45)	BIE− (n=115)	*t* ou U	*p* ‐valor
Idade (anos) a	13,6±1,6	14,5±1,5 **	4,05	0,00
Massa corporal (kg)	65,0±15,2	74,5±18,3 **	−3,09	0,00
Estatura (cm)	160,4±9,4	165,5±8,9 **	−3,18	0,00
IMC (kg/m^2^)	25,3±5,5	27,0±5,3	−1,88	0,06
IMC escore‐z	1,6±1,3	1,9±1,2	−1,30	0,19
CA (cm)	84,6±13,6	84,9±13,9	1,57	0,12
VEF_1_ (L)	2,9±0,5	3,4±0,6 **	−4,28	0,00
VEF_1_ (% predito)	95,4±10,8	102,0±18,8 *	−2,21	0,02
CVF (L)	3,4±0,7	3,9±0,8 **	−3,57	0,00
CVF (% predito)	102,3±12,2	106,0±13,4	−1,61	0,10
VEF_1_/CVF (%) a	86,7±10,3	88,5±7,0 *	2,01	0,04

* *p* <0,05;

** *p* <0,01.

a Variáveis que não apresentaram distribuição normal.

IMC, índice de massa corporal; CA, circunferência abdominal; VEF_1_, volume expiratório forçado no 1° segundo; CVF, capacidade vital forçada; VEF_1_/CVF, razão entre VEF_1_ e CVF.Valores expressos em média ± desvio padrão.

Com relação aos polimorfismos do gene do *ADRB2*, não foram encontradas diferenças significativas para o alelo 27 e para os genótipos do polimorfismo *Arg16Gly* e *Gln27Glu* entre os grupos BIE+ e BIE− (*p*=0,26; *p*=0,97 e *p*=0,43, respectivamente). Porém, foi encontrada uma tendência à significância estatística para maior proporção de polimorfismo do alelo 16 entre o grupo BIE+ comparado com o BIE− (*p*=0,08) (tabela 2).

**Tabela 2:** Frequência dos alelos e genótipos do gene *ADRB2* entre os grupos broncoespasmo induzido pelo exercício (BIE) presente (+) e ausente (−)

		BIE+	BIE−	*X* ^*2*^	*p* ‐valor
*Alelos*					
	Arg	30 (37,5%)	103 (48,6%)	2,87	0,08
	Gly	50 (62,5%)	109 (51,4%)		
	*Total*	80 (100%)	212 (100%)		
	Gln	47 (69,1%)	140 (69,3%)	0,001	0,97
	Glu	21 (30,9%)	62 (30,7%)		
	Total	68 (100%)	202 (100%)		

Genótipos					
	ArgArg	7 (17,5%)	28 (26,4%)	2,64	0,26
	ArgGly	16 (40,0%)	47 (44,4%)		
	GlyGly	17 (42,5%)	31 (29,2%)		
	*Total*	40 (100%)	106 (100%)		
	GlnGln	19 (55,9%)	51 (50,5%)	1,68	0,43
	GlnGlu	9 (26,5%)	38 (37,6%)		
	GluGlu	6 (17,6%)	12 (11,9%)		
	Total	34 (100%)	101 (100%)		

*Arg* , arginina; *Gly* , glicina; *Gln* , glutamina; *Glu* , ácido glutâmico; valores expressos em frequência absoluta e relativa.

O percentual de queda máxima do VEF_1_ apresentou correlação moderada com a presença de asma (rho=0,47; *p*<0,01). Entretanto, para as variáveis IMC escore-z (rho=0,01), CA (rho=0,20), polimorfismo *Arg16Gly* (−0,01) e *Gln27Glu* (rho=−0,07) não foram encontradas correlações significativas.

## Discussão

O objetivo do presente estudo foi determinar a influência dos polimorfismos no gene *ADRB2* no desencadeamento de BIE em adolescentes. Não foram encontradas diferenças significativas nas frequências para o alelo 27 e para os genótipos do alelo *Arg16Gly* e *Gln27Glu* no grupo BIE+ comparado com o BIE−, o que refuta, em parte, nossa hipótese inicial. Porém, verificou-se uma tendência à significância estatística para uma maior frequência do alelo 16 no grupo BIE+ comparado com o BIE−. Esse achado pode ser considerado um indício da relação entre polimorfismos no gene *ADRB2* e a presença de BIE.

Os indivíduos com diagnóstico de BIE+ apresentaram função pulmonar reduzida em relação aos BIE−, com exceção da CVF (% do predito), que não diferiu entre os dois grupos.^23^ Por outro lado, estudos anteriores não identificaram tais diferenças ao avaliar adolescentes obesos,^24^ obesos asmáticos^3^ e com rinite.^25^ Essas divergências podem ser justificadas pelas diferenças na idade e na estatura inicial dos grupos na presente pesquisa.

O percentual de queda máxima VEF1 apresentou correlação moderada com histórico de asma, o que difere do resultado encontrado por Cichalewski *et al*.^26^ As diferenças metodológicas quanto ao diagnóstico do BIE podem explicar essa discrepância, visto que na presente pesquisa foi feito teste de broncoprovocação em esteira e, no estudo de Cichalewski *et al*.,^26^ foi feito 45 minutos após uma aula de educação física. Por outro lado, ambas as pesquisas não identificaram relação do BIE com IMC.

Não foram encontrados estudos anteriores que avaliassem a frequência dos polimorfismos *ADRB2* em indivíduos com e sem BIE. Nesta pesquisa, a frequência do alelo *Gly16* foi de 62,5% e 30,7% para o alelo *Glu27*. A proporção do primeiro foi mais elevada do que em estudo feito em asmáticos que encontrou 46,6%.^8^ Entretanto, é semelhante aos achados para a população em geral (61%).^27^ A frequência do alelo *Glu27* foi semelhante entre os estudos.^8^^,^ 27

Estudo feito por Snyder *et al*.^28^ observou, em adultos saudáveis, que aqueles com o genótipo *Arg16Arg* e *Gly16Gly* têm respostas similares à broncodilatação durante o exercício. Entretanto, após o término da prova, os indivíduos homozigotos para o alelo *Arg16* retornam de forma mais rápida aos valores basais ao ser comparados com os homozigóticos *Gly16*. Os autores justificam tal achado por uma possível dessensibilização do gene *ADRB2* nessa população. Esses resultados diferem dos encontrados neste estudo, no qual não se notaram diferenças na frequência entre os genótipos, o que pode realmente confirmar não haver influência desse gene nos indivíduos com BIE.

Os polimorfismos do gene *ADRB2* podem estar relacionados com a asma principalmente por estar associados a maior sensibilidade nas vias aéreas.^8^ A hiper-responsividade brônquica é uma das características primordiais da asma e pode ser desencadeada por diversos fatores, dentre eles o exercício.^29^ Estudo feito por Fukui *et al*.^30^ mostrou que os indivíduos com maior responsividade ao teste provocado por metacolina apresentavam polimorfismo no códon 16. O BIE pode ser considerado como uma resposta exacerbada das vias aéreas.^1^ Assim, esperava-se que os polimorfismos apresentassem influência na resposta broncoconstritora ao exercício, o que acabou não ocorrendo.

O desenho transversal do estudo limita as relações de casualidade entre as variáveis. Outra questão a destacar como fator limitante é o baixo número de participantes para as análises de polimorfismos genéticos, o que leva à interpretação cautelosa dos achados da pesquisa. Sugerem-se estudos com maior número de indivíduos para confirmar a associação entre a presença do alelo *Gly*16 e a manifestação de BIE. Há necessidade de novas investigações com o controle das limitações citadas e com o uso da espirometria nos tempos 3; 5; 10 15 e 30 minutos após teste de broncoprovocação por exercício, para evitar possíveis erros de diagnóstico do BIE.

Conclui-se que a presença de polimorfismos associados ao alelo *Glu27* e aos genótipos *Arg16Gly* e *Gln27Glu* não influenciou na manifestação de BIE. Porém, a tendência estatística observada para uma maior frequência do alelo *Gly16* nos indivíduos com a presença de BIE pode ser considerada um indício da influência de polimorfismos no gene *ADBR2* no BIE em adolescentes.
